# Experience of older adults using smart devices and mHealth apps for proactive health: a descriptive qualitative study based on the technology acceptance model

**DOI:** 10.3389/fpubh.2026.1856438

**Published:** 2026-06-30

**Authors:** Huafang Li, Chunlan Liu, Qian Zhang, Chengjing Yang, Xiaorong Hu, Xiangying Yang

**Affiliations:** The Geriatrics Department of Affiliated Hangzhou First People's Hospital, School of Medicine, Westlake University, Hangzhou, Zhejiang, China

**Keywords:** older people, proactive health, qualitative research, smart device, technology acceptance model

## Abstract

**Objective:**

This study aimed to explore older adults’ experiences of using smart devices and mHealth apps for proactive health and identify the key factors affecting their adoption and sustained engagement.

**Methods:**

The current study utilized descriptive qualitative research methodology, adopting the Technology Acceptance Model (TAM) as the theoretical framework. Purposive sampling was used to recruit older adults from a tertiary Grade A general hospital in Hangzhou. Data were collected through semi-structured interviews and analyzed using directed content analysis.

**Results:**

A total of 20 older adults in Hangzhou were interviewed for the study. Analyses yielded two themes and eight sub-themes: perceived usefulness (enhanced health awareness and self-efficacy, real-time health data monitoring and early warning, accuracy and reliability of information and convenient communication with medical professionals), and perceived ease of use (interface simplicity and operability, learning cost and learning support, privacy concerns and information security, and technical support and experience sharing).

**Conclusion:**

This descriptive qualitative study explores older adults’ experiences of using smart devices and mHealth apps for proactive health, highlighting that perceived usefulness and ease of use are key determinants of their technology adoption and sustained engagement. Future digital health tool development should align with research on older adults’ user experiences to ensure these technologies’ universality and applicability.

## Background

Population aging in China has garnered widespread global attention. As indicated in the Seventh National Population Census Bulletin issued by the National Bureau of Statistics, the population aged 65 years and older in China reached 190 million as of November 1, 2020, accounting for 13.50% of the total population. This proportion is approaching the threshold for a deeply aging society (1). Against this backdrop, the Chinese government has proposed the concept of “proactive health,” which places a core emphasis on disease prevention. This strategy advocates a fundamental shift from passive, treatment-oriented interventions to proactive, prevention-focused approaches in tackling the health challenges posed by population aging (2).

As an overarching national health strategy, proactive health should be elevated to a high priority and implemented systematically at all societal levels (3). This requires coordinated engagement from medical and health institutions to deliver proactive health screenings, continuous health monitoring, and targeted intervention services, aiming to identify and mitigate health risk factors at the source and realize whole-process health management across the life course (4). At the individual level, proactive health relies heavily on fostering personal health responsibility, whereby individuals act as primary guardians of their own well-being (5). Cultivating health literacy, acquiring evidence-based health knowledge and practical self-care skills, and maintaining consistent healthy behaviors are fundamental to enabling older adults to shift from passive medical treatment to active prevention and long-term physical and mental wellness (6).

Advanced technologies, including wireless sensor networks and electronic monitoring systems for older adult care, provide a robust foundation for the development of smart wearable systems. Integrated with miniaturized sensors, actuators, and smart textile materials, such wearable platforms enable real-time health assessment and intelligent decision support for personalized health management. To date, mainstream applications of smart wearable systems are primarily concentrated on the continuous monitoring of physiological vital signs, body motion tracking, spatial localization, and automatic fall detection and prevention (7).

Despite the substantial potential of digital health technologies, the full realization of their clinical and public health benefits remains critically constrained by the variability in users’ digital literacy levels (8). Digital literacy—encompassing the skills, knowledge, and confidence required to effectively locate, evaluate, and utilize digital health tools—represents an essential foundational competency for older adults to independently operate smart devices, understand health information, and fully benefit from proactive health management (9). Low digital literacy not only increases operational barriers but also weakens older adults’ ability to judge the credibility of health content, forming a key bottleneck hindering the widespread and equitable adoption of digital health tools (10). Importantly, digital literacy is closely intertwined with the core constructs of the Technology Acceptance Model: higher digital literacy reduces perceived operational difficulty and learning burden, thereby improving perceived ease of use; meanwhile, it enables older adults to fully understand the functional value of smart health tools, further enhancing their perceived usefulness and willingness for sustained use (11).

Existing literature and systematic reviews have extensively investigated older adults’ general usage attitudes, functional demands, and barriers toward smart health technologies (12–14). Nevertheless, several specific research gaps remain. First, most existing studies focus on general technology adoption rather than being grounded in the proactive health theoretical perspective, which is central to China’s healthy aging strategy (15). Second, relevant qualitative evidence targeting hospital-recruited older adults in the Chinese cultural and Hangzhou regional context remains insufficient; local older adults’ health values, digital literacy levels, privacy concerns, and reliance on medical services differ notably from Western populations and other domestic regions (16). Therefore, this study adopted a descriptive qualitative approach guided by TAM to explore the real experiences, facilitating factors and barriers of urban older adults in Hangzhou when using smart devices and mHealth apps for proactive health, so as to fill the above targeted literature gap and provide localized evidence for the optimization of age-friendly digital health tools.

**Figure 1:**
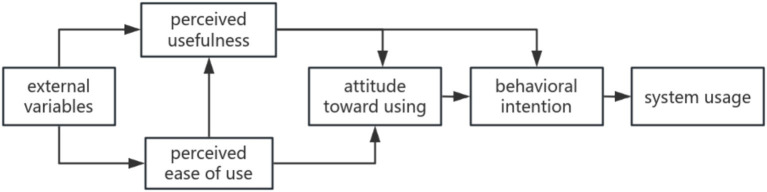
Technology acceptance model.

Derived from the Theory of Reasoned Action (TRA), the Technology Acceptance Model (TAM) was first proposed by Davis in 1986 in the field of computer and information science (17). As a classic and widely validated theoretical framework for explaining and predicting individuals’ adoption of emerging technologies, TAM has been extensively applied in research on technology use among older adults (18). Its two core constructs—perceived usefulness and perceived ease of use—can well frame older adults’ lived experiences with smart devices and mHealth apps (19). Specifically, perceived usefulness corresponds to older adults’ demands for improved health awareness, real-time health data monitoring, credible health information, and convenient communication with medical professionals; perceived ease of use reflects their practical concerns about interface design complexity, operational difficulty, learning costs, privacy and information security, as well as available technical support and peer experience sharing (20). Accordingly, adopting TAM as the theoretical framework allows this study to systematically summarize older adults’ real experiences, intrinsic needs and usage barriers in utilizing digital health tools for proactive health, and further identify the key determinants influencing their technology adoption and sustained engagement (21). The core theoretical structure of TAM is depicted in Figure 1.

## Materials and methods

This study employed a descriptive qualitative design. The TAM was used as the theoretical framework, and directed content analysis was applied as the analytical approach to explore the experiences of older adults when using smart devices and mHealth apps to pursue proactive health. Ethical approval for this study was obtained from the Academic Ethics Committee (approval code: 2025ZN281-1).

### Participants

The study was conducted in Hangzhou between September 10, 2025, and December 25, 2025. Using purposive sampling, we recruited a total of 20 participants from a tertiary Grade A general hospital in Hangzhou. Eligibility criteria were as follows: (1) aged 65 years and above; (2) experience using smart devices or mobile health applications; (3) willingness to share their experiences and skills related to the use of digital health tools; and (4) ability to provide independent judgment and communicate normally (individuals with severe mental or cognitive impairments were excluded). The final sample size was determined according to the principle of information saturation achieved during data collection. This approach is consistent with established standards in qualitative research and helps ensure the rigor, validity, and reliability of the study findings (22).

### Pre-data collection

A preliminary interview guide was developed based on a comprehensive review of the existing literature and in-depth discussions guided by the selected theoretical framework. To ensure the clinical and practical relevance of the interview content, the draft was independently reviewed by a geriatrician and a digital health program manager. A pilot interview was subsequently conducted with five participants to refine the instrument. During the pilot phase, redundant questions were deleted, and new thematic areas were added to enhance coverage of key issues. Incorporating feedback from both expert reviews and pilot testing, the interview guide was revised to align more closely with the study’s objectives. This iterative process ensured that the final tool comprehensively captured the actual experiences, needs, and perceptions of older adults, thereby strengthening the rigor and depth of the study.

### Data collection

Data were collected through face-to-face, semi-structured interviews conducted in a private room. The semi-structured interview guide was deliberately developed under the TAM theoretical framework, with each interview question conceptually mapped to the core constructs of perceived usefulness and perceived ease of use. Questions concerning health value and functional performance were designed to elicit content related to perceived usefulness; questions regarding interface operation, learning difficulty, supporting resources, privacy concerns and technical barriers were targeted to explore dimensions of perceived ease of use. Meanwhile, open-ended questions were reserved to capture unanticipated experiential views beyond the predefined TAM categories. The interview guide was designed to cover core domains grounded in the TAM framework, including older adults’usage experience and motivation of smart devices and mHealth apps, perceived usefulness and perceived ease of use, practical challenges and frustrations, available learning and technical support, as well as suggestions for function and interface optimization and willingness of recommendation. The complete version of the interview guide is provided in Supplementary material.

The interviews were conducted at a pre-agreed time in a location selected for minimal visual and auditory interference. Prior to commencement, all participants were fully briefed on the study’s purpose, methodology, and data usage. Participation was entirely voluntary, contingent upon the provision of written informed consent. To ensure confidentiality, all personal identifiers were removed, and participants were assigned codes. Participants retained the right to withdraw from the study at any time without consequence.

The sample size of participants was determined based on the principle of information saturation, which was achieved during the interview process when no new thematic content emerged and duplicate responses were consistently observed. Saturation was initially inferred from the data collected in the sixteenth interview, and four additional interviews were conducted to further verify and confirm this finding, ensuring that no new insights would be obtained with additional participants. This sampling approach is consistent with fundamental methodological principles established in prior qualitative research. All interviews were recorded in their entirety using a digital recorder; meanwhile, researchers paid close attention to participants’ body language and facial expressions to capture non-verbal cues that complemented verbal responses. Each interview had an average duration of approximately 30 min, ranging from 25 to 35 min, and detailed field notes were documented immediately after each interview to record contextual details and key non-verbal information that might not have been captured by the audio recording.

### Data analysis

Data collection and data analysis were conducted simultaneously in an iterative manner to facilitate the timely identification of emerging themes and ensure the depth and comprehensiveness of data interpretation. All interview audio recordings were transcribed verbatim within 24 h of each interview’ s completion to minimize information loss and maintain the authenticity of participants’ expressions. Given that the current study was guided by the TAM, the collected qualitative data exhibited a relatively structured nature, aligned with the theoretical constructs of the model. Therefore, the research team adopted a directed content analysis approach to systematically capture, organize, and interpret participants’ opinions, experiences, and perceptions regarding the use of smart devices and mHealth apps for proactive health. To avoid omitting diverse experiential data and prevent forcing raw information into a rigid theoretical framework, we followed a rigorous two-step analytical strategy throughout the coding process: First, all original textual materials were thoroughly coded line by line using an inductive thematic approach, without prior restriction to TAM categories. Units consistent with perceived usefulness and perceived ease of use were categorized into the predefined TAM framework. Second, any emerging codes and narratives that did not fit the established TAM constructs were independently retained, labeled, and documented as supplementary experiential reflections. These uncategorized data were fully preserved in the raw dataset and field notes rather than being discarded, ensuring the integrity, credibility and transferability of the qualitative findings. All coding, classification and thematic analysis procedures were performed using NVivo 14 qualitative data analysis software. This study was reported in accordance with the COREQ (Consolidated Criteria for Reporting Qualitative Research) checklist to ensure transparent and comprehensive presentation of all key information. The specific steps of this analytical process were as follows:

1. Identification of the unit of analysis: Sentences and paragraphs relating to behaviors, perceptions, and experiences associated with the use of smart devices and mHealth apps for proactive health were identified as the core units of analysis. This step was critical for defining the specific content and scope of the subsequent analysis, ensuring that the research focus remained aligned with the study’s objectives.
2. Iterative review and reading of raw data: The research team repeatedly reviewed and read the verbatim transcripts and field notes to gain a thorough, holistic understanding of the textual content, including the contextual nuances and implicit meanings embedded in part
3. Creation of specific categories of units of analysis: These primary categories were deductively predefined based on the TAM theoretical framework, the study’s research questions, and core objectives, ensuring that the analytical structure was theoretically grounded and research-driven. Within each TAM-based primary category, we further adopted inductive thematic analysis to extract diverse subthemes from raw data.
4. Coding and categorization: Key ideas, concepts, and experiences contained in the raw data were coded using a predefined coding scheme derived from the theoretical framework. We first conducted line-by-line open coding for all text fragments. Codes with similar content or thematic relevance were then grouped into the predefined primary categories. Within each category, we further refined and summarized codes to generate specific sub-themes, which facilitated in-depth analysis and synthesis of the textual data.
5. Interpretation and analysis of results: The coded themes and sub-themes were analyzed in depth to explore the underlying patterns and relationships among participants’ experiences. Furthermore, the study’s findings were critically evaluated to discuss their implications for real-world practice and directions for future research.

### Quality control methods

To ensure methodological rigor, the interviewer underwent systematic training in qualitative research methodology and was supervised by a senior expert in the field. The researcher communicated with interviewees after each interview to confirm findings, reflected and summarized in a timely manner, and adjusted and improved the interview outline to ensure the accuracy and completeness of the data.

## Results

A total of 20 participants were recruited for this study, including 9 males and 11 females, with ages ranging from 65 to 88 years. Detailed demographic characteristics of the participants are presented in Table 1. Through systematic data analysis, two overarching themes and eight sub-themes were identified, and their specific details are summarized in Table 2.

**Table 1:** Demographic characteristics participant.

Participant	Gender	Age (years)	Education level	Living arrangements	Types of used devices
A	Male	66	Primary school	Live with others	Smart bracelet
B	Male	70	Bachelor’s degree	Live with others	Health information app
C	Female	78	Senior high school	Live with others	Online medical consultation app
D	Female	65	Senior high school	Live alone	Smart watch
E	Female	71	Primary school	Live with others	Health information app
F	Female	71	Bachelor’s degree	Live with others	Smart glucometer
G	Female	77	Primary school	Live with others	Health information app
H	Male	69	Senior high school	Live alone	Online medical consultation app
I	Female	88	Primary school	Live with others	Smart watch
J	Male	69	Bachelor’s degree	Live with others	Smart watch
K	Female	75	Bachelor’s degree	Live alone	Smart bracelet and online medical consultation app
L	Male	88	Bachelor’s degree	Live with others	Health information app
M	Female	85	Primary school	Live with others	Smart glucometer and Health information app
N	Male	79	Senior high school	Live with others	Online medical consultation app
O	Female	74	Bachelor’s degree	Live alone	Smart watch
P	Male	74	Senior high school	Live with others	Smart bracelet and online medical consultation app
Q	Male	72	Bachelor’s degree	Live alone	Smart watch and Online medical consultation app
R	Female	70	Primary school	Live alone	Health information app
S	Male	76	Senior high school	Live with others	Smart watch
T	Female	76	Primary school	Live with others	Smart watch

**Table 2:** Themes and sub-themes of the study.

Themes	Sub-themes
1. Perceived usefulness	1. Enhanced health awareness and self-efficacy
	2. Real-time health data monitoring and early warning
	3. Accuracy and reliability of information
	4. Convenient communication with medical professionals
2. Perceived ease of use	1. Interface simplicity and operability
	2. Learning cost and learning support
	3. Privacy concerns and information security
	4. Technical support and experience sharing

### Perceived usefulness

Older adults’ perceptions of the usefulness of smart devices and mHealth applications constitute holistic and ongoing evaluations that persist throughout the entire period of tool usage. Older individuals tend to continuously assess whether a specific digital health solution can fulfill its intended functions and achieve anticipated health outcomes, as well as whether it aligns with their actual health needs, daily routines, and functional capacities. When the use of these smart devices and mHealth apps better addresses their health demands, delivers greater convenience, and provides more tangible benefits compared with traditional health management approaches, participants expressed a stronger willingness to accept the technology and acknowledge its practical usefulness in supporting their proactive health management.

### Enhanced health awareness and self-efficacy

Engagement with smart devices and mHealth apps enabled older adults to develop a more systematic understanding of their health status and cultivate a stronger sense of health self-management. Through regular interaction with these tools, participants gradually transitioned from passive health recipients to proactive managers, thereby improving both their health awareness and self-efficacy in maintaining well-being.

Respondent D: “I used to only go to the hospital when I felt unwell, but now I check my health indicators on my watch every day. I know more about my body and feel more confident in taking care of myself. It makes me feel in control of my own health (laughs).”

Another key aspect of enhanced health awareness and self-efficacy was the sense of empowerment derived from active engagement with smart devices and mHealth apps. Participants reported that regular interaction with these tools reduced their anxiety about health issues and increased their confidence in making informed health decisions, as they could access real-time feedback and actionable advice tailored to their individual needs.

Respondent Q: “I used to get nervous every time I felt a little uncomfortable, worrying that something was seriously wrong. Now, I can check my vital signs with my smart watch and see if they are within the normal range. This not only makes me more aware of my health status but also gives me the confidence to handle small health issues on my own, without rushing to the hospital.”

### Real-time health data monitoring and early warning

A core perceived usefulness of smart devices and mHealth apps was their ability to monitor health data in real time and issue timely early warnings, which aligned closely with older adults’ needs for proactive health management. These tools continuously captured key physiological indicators, allowing participants to track changes in their health status dynamically and detect potential abnormalities at an early stage.

Respondent A: “My smart bracelet monitors my blood pressure every hour and sends me a reminder if it’s too high. Last month, it warned me that my blood pressure was abnormally elevated, so I adjusted my medication and went to the doctor for a check-up. The doctor said that catching it early prevented a more serious problem (nods).”

### Accuracy and reliability of information

The perceived usefulness of smart devices and mHealth apps among older adults was strongly contingent on the accuracy and reliability of the health data and information they provided. Participants emphasized that trustworthy measurements and credible health content were fundamental prerequisites for their willingness to adopt and persist in using these tools, as inaccurate information could lead to misjudgments about their health and potentially harmful decisions.

Respondent T: “I compared the blood pressure readings from my smart watch with the ones taken at the community hospital, and they were almost the same. That’s why I trust this device—if the data wasn’t accurate, I would have stopped using it long ago. Reliable information is the most important thing for us.”

In addition to data accuracy, older adults also valued the reliability of health advice and educational content provided by mHealth apps. They preferred apps that offered information from authoritative sources and avoided misleading or exaggerated content, as this enhanced their confidence in the tool’s utility for proactive health management.

Respondent Q: “Some apps have a lot of health tips, but I do not know which ones are true. This app only provides advice from real doctors, and it explains things in simple terms that I can understand. I know the information is reliable, so I follow the suggestions it gives me to take care of my health.”

### Convenient communication with medical professionals

Smart devices and mHealth apps significantly improved the perceived usefulness by facilitating convenient, efficient communication between older adults and medical professionals, addressing the barriers of distance, time, and mobility that often hinder access to healthcare services. These tools allowed participants to share health data, consult with doctors, and receive professional advice without the need for in-person visits, thereby enhancing the accessibility of healthcare support for proactive health management.

Respondent C: “I live in a rural area, and the nearest hospital is an hour’s drive away. Before using the mHealth app, I had to ask my son to take me to the hospital every time I had a question. Now, I can send my blood pressure and heart rate data to my doctor directly through the app and get a reply within a few minutes. It’s so convenient and saves me a lot of time and energy.”

### Perceived ease of use

Older adults’ perceived ease of use of smart devices and mHealth applications is a key factor influencing their adoption of these tools for proactive health management. When determining usability, they evaluate whether the technology is within their functional capabilities, whether they feel comfortable and confident during use, and whether they require extensive external support or guidance. These perceptions in turn shape how easily they can interact with the tools to engage in proactive health practices and access relevant health information.

### Interface simplicity and operability

Perceived ease of use of smart devices and mHealth apps among older adults was strongly associated with interface simplicity and operability, which are critical for their ability to independently utilize these tools for proactive health management. Older adults, often with limited digital literacy and declining visual function, prioritized interfaces with clear, large icons, simple navigation, and intuitive operations, as these features reduced operational barriers and enabled them to access health-related functions without excessive effort.

Respondent L: “The app I use has big, clear icons, and the words are large enough for me to see without my reading glasses. I just tap the icon for blood pressure, and it automatically records my data. It’s so simple that I can use it by myself, without asking my granddaughter for help every time.”

### Learning cost and learning support

The learning cost of smart devices and mHealth apps refers to the time, effort, and cognitive load required to master basic operations, and the availability of learning support were key determinants of older adults’ perceived ease of use. Participants reported that low learning costs and accessible learning support significantly reduced their resistance to adopting these tools for proactive health management.

Respondent I: “I’m 78 years old, and I learn new things slowly. But this smart watch was easy to learn. My son showed me how to check my heart rate once, and I remembered it right away. There are also simple video tutorials in the app that I can watch again if I forget, so I do not have to bother him all the time.”

Respondent E: “At first, I could not figure out how to connect the app to my blood glucose meter. But the app has a voice guide that talks me through each step —‘open the app, click connect, place the meter near the phone’. It’s like having someone teaching me. Now I can connect them by myself and check my blood sugar whenever I want, which helps me take better care of my health.”

### Privacy concerns and information security

Many older adults expressed fears about the leakage of their personal health information and personal data when using these tools, which undermined their confidence and willingness to use the tools for proactive health management.

Respondent G: “I was hesitant to use this app at first because I was worried that my blood pressure and heart rate data would be leaked to strangers. I’ve heard stories about old people’s personal information being stolen online, and I do not want my health details to be used by others.”

Older adults’ perceived ease of use was significantly enhanced when smart devices and mHealth apps provided clear privacy protection measures and transparent information usage policies. Participants reported that visible security features alleviated their privacy concerns, making them more comfortable and confident in using the tools to engage in proactive health management.

Respondent H: “I started using the app only after my son showed me that it has a password and that my health data is only visible to me and my doctor. The app also tells me clearly that it will not share my information with other companies. Knowing my privacy is protected makes it easy for me to use the app to check my health every day without worrying.”

### Technical support and experience sharing

Participants reported that timely resolution of technical issues and the ability to learn from others’ experiences reduced their frustration and increased their confidence in using the tools for proactive health management.

Respondent L: “Once my app stopped syncing my health data, and I did not know what to do. I was worried I’d lose all my records. I called the customer service number on the app, and they walked me through the steps to fix it in 5 min. Having that technical support makes me feel safe using the app, knowing I can get help if something goes wrong.”

Additionally, experience sharing with peers was another important form of support, as it provided practical, relatable guidance tailored to the needs of older adults. Participants noted that learning from others’ successes and challenges made it easier to master the tools and integrate them into their proactive health management practices.

Respondent M: “My neighbor uses the same mHealth app as me, and we often talk about how to use it. She showed me a trick to quickly find my health records, and I told her how to set up reminders for medication.”

## Discussion

### Perceived usefulness analysis

The findings of this study indicate that older adults’ perceived usefulness of smart devices and mHealth apps is notably heightened when they perceive these tools as outperforming traditional health management approaches in addressing their health-related requirements, which is consistent with findings reported a previous study by Yu et al. (23) Additionally, participants consistently reported that the ability of smart devices and mHealth apps to provide real-time health data monitoring and early warning alerts was the most valued aspect of their usefulness for proactive health. Many older adults noted that these tools allowed them to track vital signs continuously, enabling them to detect subtle changes in their health status at an early stage. This aligns with previous qualitative research by Ding, which identified “early warning alerts” as a key sub-theme of perceived usefulness among older adults using digital health tools (24). Similarly, a study on smart home-based proactive health technologies found that older adults valued the ability to anticipate health crises through regular monitoring, which enhanced their sense of control over their health (25). This finding underscores the critical role of digital tools in translating proactive health from a conceptual framework to a practical practice for older adults, who are particularly vulnerable to chronic diseases and sudden health declines.

Smart devices and mHealth apps may have a positive effect on enhancing older adults’ self-care efficacy, a core component of proactive health (26, 27). Participants reported that the personalized health recommendations, medication reminders, and health education modules provided by mHealth apps empowered them to take an active role in managing their health, rather than relying solely on periodic medical consultations (28, 29). For example, participants with chronic conditions noted that apps tailored to their specific health needs helped them adhere to treatment plans and make informed lifestyle adjustments, which in turn reduced their anxiety about health deterioration (30). This is consistent with the findings of a web-based survey study exploring smart health wearable adoption among Singaporean older adults, which was conducted based on the Self-Determination Theory and highlighted the positive impact of digital health tools on older adults’ self-care capabilities and overall quality of life (31).

Notably, older adults’ evaluations of smart devices and mHealth apps for proactive health are shaped not only by the inherent functional performance of these digital tools but also closely linked to their individual lived experiences (32). Health status, educational attainment, digital literacy, and prior technology engagement collectively exert substantial influences on their subjective assessments (33). Equally important are older adults’ psychological expectations, and an in-depth understanding of these expectations is critical to accurately interpreting their perceived usefulness of such digital health interventions (34). In practice, perceived usefulness is largely predicated on trust in the functional accuracy and reliability of smart devices and mHealth apps (35). Such trust can be easily undermined by inaccurate data or unsatisfactory operational performance during use, potentially triggering user frustration, diminished confidence, and subsequent reduced engagement or discontinuation of use, as is consistent with the findings reported by Portz et al. (36)

As emphasized by the Technology Acceptance Model, perceived usefulness of new technologies is the core driving factor for users’ adoption of such technologies, and this holds true for older adults’ acceptance of smart devices and mHealth apps for proactive health management as well (37). Developers should prioritize optimizing the functional design of these digital tools based on the perceived usefulness dimensions identified in this study, such as strengthening real-time health monitoring accuracy, refining personalized health recommendations, and simplifying operational processes to accommodate older adults’ limited perceptual, motor, and cognitive capabilities (38). Additionally, developers should establish a reliable data verification mechanism to improve the accuracy and reliability of health data, fostering users’ trust and thus reinforcing their perceived usefulness, as trust is a fundamental prerequisite for older adults to recognize the value of digital health tools (39).

### Perceived ease of use analysis

Perceived ease of use, a pivotal construct in the TAM, also serves as a critical determinant of sustained engagement with digital tools for proactive health (40). Unlike younger populations, older adults often face unique physiological and cognitive challenges, including declining visual acuity, reduced motor dexterity, and slower information processing speed, which can significantly influence their perceptions of ease of use and subsequent technology adoption behaviors (41). The findings of this study reveal that perceived ease of use is closely intertwined with older adults’ willingness to integrate smart devices and mHealth apps into their daily proactive health routines, with participants frequently citing “simplicity of operation” and “minimal learning burden” as key factors shaping their positive evaluations (42).

Notably, social support and targeted training emerged as key facilitators of perceived ease of use among older adults in this study. Participants who received guidance from family members, healthcare providers, or community digital literacy programs reported higher levels of perceived ease of use, as they were able to overcome initial operational challenges and build confidence in using the tools (43). This is consistent with a study by Ding et al., which demonstrated that tailored digital training and social support significantly improved older adults’ perceived ease of use of smart health technologies, thereby promoting long-term engagement (24). In contrast, participants without such support often reported feeling overwhelmed by the technology, leading to negative perceptions of ease of use and reduced adoption. This highlights the importance of contextual factors in shaping older adults’ perceived ease of use, beyond the inherent design of the digital tools themselves.

The findings of this study indicate that technical support and experience sharing among older adults themselves significantly enhance their perceived ease of use of digital health tools. Such peer-to-peer support often involves the exchange of practical operational tips, problem-solving strategies, and first-hand usage experiences that are highly relevant to the specific needs and challenges of older adults (44). This peer support not only reduces the learning burden and operational anxiety associated with digital tool use but also fosters a sense of mutual assistance and confidence, making the process of learning and using smart devices and mHealth apps less intimidating. This finding is consistent with previous research by Jokisch et al. (45). Thus, the mutual support and experience exchange among older adults serve as a valuable complement to formal digital literacy training and family support, further strengthening their perceived ease of use and promoting sustained engagement with digital health tools for proactive health management.

From a TAM perspective, integrating privacy protection design into smart devices and mHealth apps further strengthens their acceptance: as TAM emphasizes, perceived ease of use and usefulness are core to adoption, and robust privacy safeguards (e.g., transparent data collection policies, secure storage) alleviate older adults’ concerns about health data leakage, thereby boosting their willingness to engage with these tools and reinforcing the positive feedback loop between ease of use and usefulness, ultimately facilitating their proactive health management through sustained digital tool adoption (46).

### Cross-cultural comparison and practical implications for digital health tool design

Cultural and regional contexts are well-recognized critical factors shaping older adults’ acceptance and use of digital health technologies. For qualitative research focusing on user experience, discrepancies in social norms, healthcare systems, family culture and national health strategies will inevitably lead to varied research findings across different regions and populations. Accordingly, this study’s findings were further compared with two representative Western studies from Spain and the UK to clarify cross-cultural similarities and differences in older adults’ mHealth adoption and needs. The Spanish study highlighted that retirement-age adults preferred intuitive interfaces, simplified operations, personalized goal-setting, tailored health content, and positive feedback mechanisms, while low digital literacy, technology dependence concerns, and the need for localized culturally adapted content were major barriers (47). The UK study further indicated that app interface design directly affects perceived ease of use; perceived usefulness was undermined by unreliable health information, privacy leakage risks, and unclear practical benefits. Aging generation gaps, complex operational workload, and potential economic loss were also key adoption barriers among British older adults (48).

Consistent with Western counterparts, older adults in Hangzhou also valued concise interface layout, easy operability, reliable health data, and privacy protection, with low digital literacy and high learning costs being common shared obstacles. Nevertheless, distinct cultural and contextual differences existed. Chinese older adults relied more on formal medical institutions and tended to connect daily monitoring data with offline hospital follow-up. They depended heavily on family guidance and community peer experience sharing, rather than pursuing persuasive design or in-app social functions emphasized in the Spanish sample. Meanwhile, driven by the national proactive health strategy, local older adults showed stronger demand for real-time physiological monitoring and early warning functions, and their privacy concerns were more focused on sensitive health data leakage compared with British participants’ worries about information credibility and economic risks.

The present qualitative findings offer in-depth practical implications for the future design and development of age-friendly digital health interventions targeting older adults. In terms of functional feature optimization, developers should prioritize embedding high-precision real-time physiological monitoring, intelligent early warning reminders, authoritative and simplified health knowledge push, and one-click online consultation modules to fully meet older adults’ demands reflected in the perceived usefulness dimension (49). Meanwhile, medication reminder, health record automatic storage, and personalized health suggestion functions should be further refined to strengthen older adults’ health self-management efficacy. From the perspective of user experience and interface design, digital health tools for older adults should adhere to the principles of simplicity and accessibility. Specifically, designers should adopt large-font display, enlarged icon layout, streamlined navigation logic, and reduce redundant operational steps to lower use barriers (50). Additionally, built-in voice guidance, visualized operational tutorials, and accessible one-click technical support entry should be incorporated to reduce learning costs. Considering older adults’ widespread privacy concerns, transparent data privacy agreements, independent password protection, and encrypted health data storage should be set as standard configurations to enhance information security and user trust (51). Furthermore, community-based peer experience sharing mechanisms and regular offline digital literacy training should be combined with intelligent device functions, forming a complementary support system to help older adults continuously improve their ability to engage in proactive health management via digital health tools (52).

### Study limitations

This study has several limitations. First, the sample may not have comprehensively represented all subgroups of older adults, potentially affecting the generalizability of the findings. Since this study aimed to explore real usage experiences of digital health tools, all participants were existing users of smart devices and mHealth apps, who generally had relatively higher digital literacy than the broader older adult population. Data saturation was fully achieved during data collection, as no new themes emerged in later interviews, which meets the standard requirements for qualitative research. In addition, the functions of devices and applications discussed in the interviews were unevenly distributed: besides physiological monitoring, participants also used medication reminders and health record management functions. Nevertheless, physiological monitoring remained the most frequently mentioned function, while exercise tracking and other auxiliary functions were less addressed. This imbalance further limits the transferability of the results. Future studies should expand recruitment to capture a fuller range of experiences with smart devices and mHealth apps for proactive health, and pay more attention to under-discussed functional scenarios.

Second, this study may have theoretical bias due to the adoption of a fixed TAM framework. Some extracted themes cannot perfectly align with TAM’s core constructs. For example, privacy and information security concerns were classified under perceived ease of use, as complex privacy and security settings created extra operational burdens for older users. We recognize this is an extended application beyond the original scope of perceived ease of use. To reduce such bias and guarantee data integrity, we followed a strict protocol to handle emergent themes. During analysis, we performed open coding for all raw transcripts first, without rigidly adhering to the predefined TAM categories. For content closely linked to device operation, we allocated it to appropriate TAM dimensions after comprehensive consideration. Any themes that could not be reasonably incorporated into the two core constructs were separately documented and preserved as supplementary results, instead of being arbitrarily categorized or removed. This method helped balance theoretical guidance and the objectivity of qualitative findings.

## Conclusion

In conclusion, this descriptive qualitative study explores older adults’ experiences of using smart devices and mHealth apps for proactive health, highlighting that perceived usefulness and ease of use are key determinants of their technology adoption and sustained engagement. Future development of digital health tools should be advanced in tandem with research on older adults’ user experiences to ensure the universality and applicability of these technologies.

## Data Availability

The raw data supporting the conclusions of this article will be made available by the authors, without undue reservation.
