# Supplementary material for: Experience of older adults using smart devices and mHealth apps for proactive health: a descriptive qualitative study based on the technology acceptance model
Source: Front Public Health. 2026 Jun 30;14:1856438. doi: 10.3389/fpubh.2026.1856438 (PMC13365344; doi:10.3389/fpubh.2026.1856438)
Supplement: Supplementary file 1 [file Data_Sheet_1.DOCX]

**Interview Guide**

1. Could you briefly describe the smart devices and mHealth apps you have used for proactive health management?
2. What motivated you to start using these smart devices and mHealth apps for proactive health?
3. How would you evaluate the usefulness of these smart devices and mHealth apps in supporting your proactive health practices?
4. How easy or difficult do you find it to use these smart devices and mHealth apps?
5. What challenges or frustrations have you encountered when using these smart devices and mHealth apps to support your proactive health?
6. Have you received any support when using these smart devices and mHealth apps?
7. Based on your current experience, what improvements would you suggest for smart devices and mHealth apps to better meet your proactive health needs?
8. Would you recommend these smart devices and mHealth apps to other older adults who want to engage in proactive health management?
